# Circadian rhythm parameters differentiate euthymic, manic and depressive mood states in bipolar disorders – an explorative pilot study

**DOI:** 10.1186/s40345-025-00396-5

**Published:** 2025-10-27

**Authors:** J. Clemens, E. Mühlbauer, I. Reinhard, M. Bauer, A. B. Neubauer, P. Ritter, V. M. Ludwig, W. E. Severus, U. W. Ebner-Priemer, S. E. Schmitz

**Affiliations:** 1 Department of Psychiatry and Psychotherapy, Faculty of Medicine and University Hospital Carl Gustav Carus, Dresden University of Technology, Fetscherstraße 74, 01307 Dresden, Germany; 2 Mental mHealth Lab, Institute of Sport and Sport Sciences, Karlsruhe Institute of Technology, Karlsruhe, Germany; 3 Asklepios Klinik Nord - Ochsenzoll, Hamburg, Germany; 4 Department of Biostatistics, Medical Faculty Mannheim, Central Institute of Mental Health, University of Heidelberg, Mannheim, Germany; 5 Department of Psychiatry and Psychotherapy, Medical Faculty Mannheim, Central Institute of Mental Health, University of Heidelberg, Mannheim, Germany; 6 Institute of Psychology, RWTH Aachen University, Aachen, Germany; 7 Institute of Psychiatry, King’s College London, Psychology & Neuroscience (IoPPN), London, England, UK

**Keywords:** Circadian rhythm, Bipolar disorders, Ecological momentary assessment, Actigraphy, Early warning signs, Physical activity

## Abstract

**Background:**

Bipolar disorders (BD) pose significant therapeutic health challenges due to recurrent and largely unpredictable depressive and (hypo)manic episodes. Traditional self-report methods for symptom monitoring are limited by their dependence on patient adherence which is frequently diminished during symptomatic phases. Circadian movement patterns, measured via actigraphy, have emerged as promising digital biomarkers for distinguishing mood states in BD. This study examined the utility of circadian rhythm parameters in differentiating euthymic, depressive, and (hypo)manic states.

**Methods:**

This study analyzed data from 27 BD patients (mean age = 46 years, 16 female) monitored over 12 months as part of the BipoSense project. Wrist-worn accelerometers continuously recorded physical activity, while mood state was assessed using daily self-reports and biweekly expert evaluations. Circadian rhythm parameters included interdaily stability (IS), intradaily variability (IV), mean activity difference (MeanDiff), and circadian form difference (FormDiff). IS and IV reflect rhythm stability and fragmentation, while MeanDiff and FormDiff quantify overall activity and deviations in circadian rhythm form. Multilevel models were used to predict categorical mood states (depressive, (hypo)manic, euthymic) and dimensional symptom severity.

**Results:**

Physical activity data from 23 patients yielded 2,669 valid days for analysis. In multilevel logistic models, lower MeanDiff (*B* = –.02, *P* < .001), reflecting reduced overall activity, lower IS (*B* = –.80, *P* = .009), indicating less stable circadian rhythms, and higher FormDiff (*B* = .03, *P* < .001), denoting a more rigid circadian activity pattern, were significantly associated with increased odds of depressive days compared to euthymic days. Conversely, higher MeanDiff (*B* = .02, *P* = .007) was linked to higher odds of (hypo)manic days. Dimensional linear mixed models showed a similar pattern: lower MeanDiff (*β* = –.11, *P* < .001), IS (*β* = –.06, *P* = .001), and IV (*β* = –.06, *P* = .002), together with higher FormDiff (*β* = .10, *P* < .001), predicted increased depressive symptom levels. Conversely, higher MeanDiff (*β* = .10, *P* < .001), IS (*β* = .04, *P* = .024), IV (*β* = .07, *P* < .001), and lower FormDiff (*β* = –.07, *P* = .001) were associated with heightened (hypo)manic symptoms.

**Conclusions:**

Circadian rhythm parameters can effectively differentiate mood states in BD, highlighting their potential as clinical markers for episode transitions. Although the study was explorative by nature, the findings emphasize the potential value of integrating circadian biomarkers into digital phenotyping for mood state monitoring. Future studies should explore extended monitoring periods, larger samples, and real-time feedback systems to improve early intervention and personalized treatment strategies in BD.

**Supplementary Information:**

The online version contains supplementary material available at 10.1186/s40345-025-00396-5.

## Introduction

Bipolar disorders (BD) pose a major public health challenge, often manifesting as a recurrent or chronic condition (Carvalho et al. 2020; Grande et al. 2016). Predicting and preventing new episodes is therefore a key treatment objective. Traditional charting methods (Bauer et al. 2023), however, rely heavily on patient adherence and motivation to complete forms consistently over extended periods, and their reliability often diminishes as patients’ insight into their illness declines, especially during manic episodes. To enable timely intervention and support ongoing treatment and secondary prevention, there is a critical need for objective, unobtrusive, and continuous monitoring that can detect emerging symptoms and episode onset within patients’ everyday environments (Morriss et al. 2007).

The gold standard for objective, unobtrusive, and continuous monitoring is Ambulatory Assessment (AA), a technology that combines passive sensing via wearables and smartphones – often referred to as digital phenotyping – with active assessments like e-diaries. This approach, marked by a) minimal disruption to daily routines, b) long-term continuous measurement over months and even years, and c) real-time analysis and feedback capabilities, offers promising potential for tracking mood disorder symptoms through smartphone sensors or actigraphy wearables (Ebner-Priemer et al. 2020; Friedmann et al. 2022). While automated tracking of digital phenotypes has become a highly desired method (Organization 2019) and has shown success in monitoring symptomatology more broadly (Reichert et al. 2020; Santangelo et al. 2020; Yerushalmi et al. 2021), progress in detecting upcoming episodes in BD remains limited (Anmella et al. 2022). Some observational studies confirm associations between digital phenotypes and symptomatology, whereas others reveal contradictory findings (Beiwinkel et al. 2016; Gershon et al. 2016; Grunerbl et al. 2015). Overall, explained variance in psychopathology remains very low (Ebner-Priemer et al. 2020). Randomized controlled trials (RCTs) using digital phenotyping as a preventive tool for BD episodes also yielded no significant results on primary outcomes (Faurholt-Jepsen et al., 2014, 2016). Four challenges might explain large heterogeneity in findings and limited success: (1) digital phenotypes with low validity, (2) limited longitudinal long-term assessments to capture emerging episodes, (3) neglected high-frequency psychopathological gold-standard expert ratings, and (4) missing time sensitive indices.

In terms of validity, Wadle and Ebner-Priemer (2023) have pointed out that most digital phenotyping studies rely on easily accessible technological parameters, such as steps per day. Less often used are indices which are harder to acquire but show a close alignment to psychopathology. Disturbances in circadian rhythms and variations in psychomotor activity are promising examples, as they are core indicators of BD symptomatology (Faurholt-Jepsen et al. 2016; Jones et al. 2005; Krane-Gartiser et al. 2014) and are considered vulnerability factors during subsyndromal periods (Jones et al. 2005; Murray et al. 2020). Specifically, in bipolar depression, psychomotor retardation and sleep disturbances (among the seven criterion B symptoms) are key symptoms, whereas in (hypo)manic episodes, increased goal-directed activity or energy, paired with an elevated, expansive, or irritable mood (criterion A) are required (American Psychiatric Association, 2018). Taking the validity argument seriously, it is not surprising that studies which use broad measures of activity (low validity), such as GPS- or cell-tower-based movements (Beiwinkel et al. 2016; Braund et al. 2022; Faurholt-Jepsen et al., 2014, 2016, 2021), show less promising findings compared to studies tracking circadian rhythm using wearables (Lim et al. 2024; Ortiz et al. 2025).

To investigate whether circadian movement patterns change from euthymic to symptomatic states, **longitudinal within-subject studies** are needed. However, acording to recent reviews (Scott et al., 2020; Panchal et al. 2022) most studies have employed cross-sectional designs. They either compare BD patients with healthy controls or compare a group of patients during depressed states with a different group of patients during manic states (Busk et al. 2020; Faurholt-Jepsen et al. 2012; Hatonen et al. 2008; Jones et al. 2005; Krane-Gartiser et al. 2014; Palmius et al. 2017; Yerushalmi et al. 2021; Zhang et al. 2023). While valuable, these between-subject designs do not provide insights how patterns change from euthymic to symptomatic states. However, the desired longitudinal within-subject studies also encounter limitations, especially in their observation period (Kunkels et al. 2021), with most studies spanning only a few months (three months or less: Beiwinkel et al. 2016; Braund et al. 2022; Ferrand et al. 2022; Gershon et al. 2016; Grunerbl et al. 2015; Walsh et al. 2022, 2023), thereby revealing a limited number of emerging episodes (Ebner-Priemer et al. 2020). Undoubtedly, to effectively assess the potential of actigraphy in predicting episodes, a sufficient number of emerging episodes is essential.

Moreover, many studies lack **temporal precision** in psychopathological assessments. Most studies rely on monthly diagnostic interviews at best, which limits the ability to precisely capture the onset of new episodes (Ebner-Priemer et al. 2020) and they often use dimensional self-report measures instead of **gold-standard structured clinical interviews**. For example, the impressive study by Lim et al. (2024), which monitored 111 patients with BD and achieved on average 267 days of wearable data, used gold-standard structured clinical interviews to confirm clinical status. Unfortunately, these were conducted only every three months in retrospect, questioning the temporal precision needed to predict onset at a day level.

Earlier wake times or delayed bedtimes are characteristics of altered circadian patterns in BD but are unfortunately not covered in standard circadian rhythm indices. The most often used actigraphy-based indices are interdaily stability (IS) and intradaily variability (IV) (van Someren et al. 1996; 1999), depicting reduced stability in the activity rhythm (as indexed by IS) and greater rhythm fragmentation (as indexed by IV). As they merely average daily activity and do not investigate the circadian form, we incorporate **time-sensitive indices** of circadian rhythm.

In conclusion, there is a clear need for studies featuring long-term within-subject assessments (e.g., 12 months) capturing a sufficient number of emerging episodes. Such studies should incorporate a) frequent gold-standard psychopathological assessments ensuring appropriate temporal precision, b) valid parameters, and c) time-sensitive indices to produce more robust results. To investigate whether circadian movement patterns differ between euthymic and symptomatic states, we continuously monitored actigraphy data from 27 BD patients over 12 months, collecting both dimensional and categorical expert ratings every 14 days, along with daily self-ratings of psychopathological status. Our study aimed to determine whether circadian movement patterns could effectively differentiate asymptomatic (euthymic) days from symptomatic (depressive or manic) days in BD, based on categorical expert ratings, and how these patterns relate to dimensional symptom severity ratings.

## Methods

Data for this study were collected as part of the BipoSense project (Ebner-Priemer et al. 2020), designed to distinguish depressive, euthymic, and (hypo)manic mood states based on digital phenotypes. Participants’ actigraphy data were continuously monitored over a 12-month period, complemented by the collection of digital phenotypes (not analyzed in this manuscript). Daily self-reported assessments of psychopathology were supplemented by expert ratings and interviews every two weeks, providing 26 assessments per participant. Recruitment took place at the Department of Psychiatry at the Technical University of Dresden, Germany, with detailed study procedures outlined in Ebner-Priemer et al. (2020).

The main inclusion criteria were a confirmed BD diagnosis, with patients in full or partial remission at enrolment (DSM-5: 296.46; 296.56; 296.89; YMRS score ≤ 12 and MADRS score ≤ 12), and a history of at least three affective episodes in the past five years, including at least one (hypo)manic episode. This analysis includes data from 23 patients who also wore an acceleration sensor to record physical activity. Ethical approval was granted by the IRB of the University of Dresden (DE/EKSN38, reference number: 26012014).

*Psychopathological status:* A trained psychologist administered categorical and dimensional diagnostic instruments, alternately in person at the University Hospital Dresden and by telephone. The SCID-I (Section A) was used to identify current affective episodes according to DSM-5 criteria for the prior two weeks (First et al., 2015). (Hypo)manic and depressive symptoms were further assessed using the German version of the Young Mania Rating Scale (YMRS; Young et al.^1978^), the Bech-Rafaelsen Mania Rating Scale (BRMRS; Bech, et al. 1979), and the Montgomery-Åsberg Depression Rating Scale (MADRS; Montgomery & Åsberg 1979) each measuring symptoms over the previous three days. These instruments exhibit excellent reliability and validity. Additionally, patients completed daily end-of-day mood assessments using a visual analog scale (0–100) to rate their current mood from “depressed” to “elevated”, adapted from ChronoRecord (Bauer et al. 2008).

Two approaches were employed to classify daily psychopathological states as depressed, (hypo)manic, or euthymic. First, a categorical approach based on SCID-I interview data classified each day as part of a depressive, (hypo)manic or euthymic episode. (Ebner-Priemer et al. 2020) Second, we employed separate general linear mixed models to examine the effects of circadian rhythm measures on daily variation in manic and depressive psychopathological status. To capture these daily variations, we constructed two latent outcome variables using structural equation modeling (SEM). Each latent factor (mania and depression) was based on three types of indicators: (1) a categorical expert rating reflecting the presence or absence of a DSM-IV-defined affective episode on a given day, (2) dimensional expert ratings – Montgomery-Asberg Depression Rating Scale (MADRS; Montgomery and Asberg, 1979) for depressive symptoms, and Bech-Rafaelsen Mania Rating Scale (BRMRS; Bech et al. 1979) plus the Young Mania Rating Scale (YMRS; Young et al. 1978) for manic symptoms, and (3) self-ratings from end-of-the-day diaries assessing manic-depressive mood (visual analog scale “depressed” to “elevated”; 0–100; adapted from ChronoRecord, (Bauer et al. 2012). These indicators were combined into latent constructs, assuming that the observed variables reflect a common underlying psychopathological state. Model convergence was satisfactory, with scale reduction factors of 1.001 for mania and 1.003 for depression (Ebner-Priemer et al. 2020). Although more complex, this latent approach allows for greater precision by: i) enhancing temporal resolution through the integration of high-frequency data, ii) differentiating symptom severity beyond binary outcomes; and iii) reducing inflation of chance by unifying all outcome variables. For details on latent score calculation, see Ebner-Priemer et al. (2020), where structural equation modelling (SEM) in Mplus (Asparouhov et al. 2018) was used to compute these scores. SEM was performed with Bayesian estimators, default (uninformative) priors with two chains, 10,000 iterations (with the first half discarded as burn-in), and a thinning factor of 300, yielding the two latent variables “depressive” and “(hypo)manic”.

*Assessment of physical activity (PA):* PA data were recorded using a triaxial accelerometer (Move 3, Movisens GmbH, Karlsruhe, Germany, www.movisens.com) worn on the non-dominant wrist. The device captured raw acceleration data (± 8 g range, 4 m-g noise, 12-bit resolution, 64 Hz A-D rate), stored on the sensor for up to one month. The sensor was recharged weekly, and data were downloaded and cleared at each hospital visit to ensure data integrity. Initial technical issues with an earlier sensor model led to some data loss, prompting a switch to the Move 3 model.

*Analysis of PA data:* Accelerometer data were band-pass filtered (0.25 to 11 Hz), and vectorized, with mean acceleration computed over one-minute intervals (band-pass filtered euclidean norm, BFEN). A polysomnography-validated algorithm (Barouni et al. 2020) was used to classify each interval as physically active, asleep, or nonwear. Analyses were conducted using DataAnalyzer v.1.11.2 software (www.movisens.com).

*Analysis of circadian movement patterns:* To enable comparability with previous studies (e.g., Murray et al. 2020), we analyzed circadian activity using two established actigraphy-based parameters: Interdaily stability (IS) and intradaily variability (IV) (van Someren et al. 1996, 1999).

Adopted to our dataset, IS is the variance of the 1440 min of one day divided by the variance of all minutes of all euthymic days. Variance computation is based on the squared difference between each minute (of a day/of all days) and the mean of all euthymic minutes (of a day/of a subject). In short, IS compares the variance within a day with the variance of all days of the measuring period. According to Murry and colleagues (2020) a lower IS indicates reduced stability in the activity rhythm.

IV is computed as the daily mean of the squared difference between consecutive minutes divided by the variance of all euthymic minutes. IV aims to measure rhythm fragmentation, reflecting the frequency of transitions between rest and activity in a given 24-h period. According to Murry and colleagues (2020) a higher IV is interpreted as greater fragmentation of the activity rhythm.

**Fig. 1:**
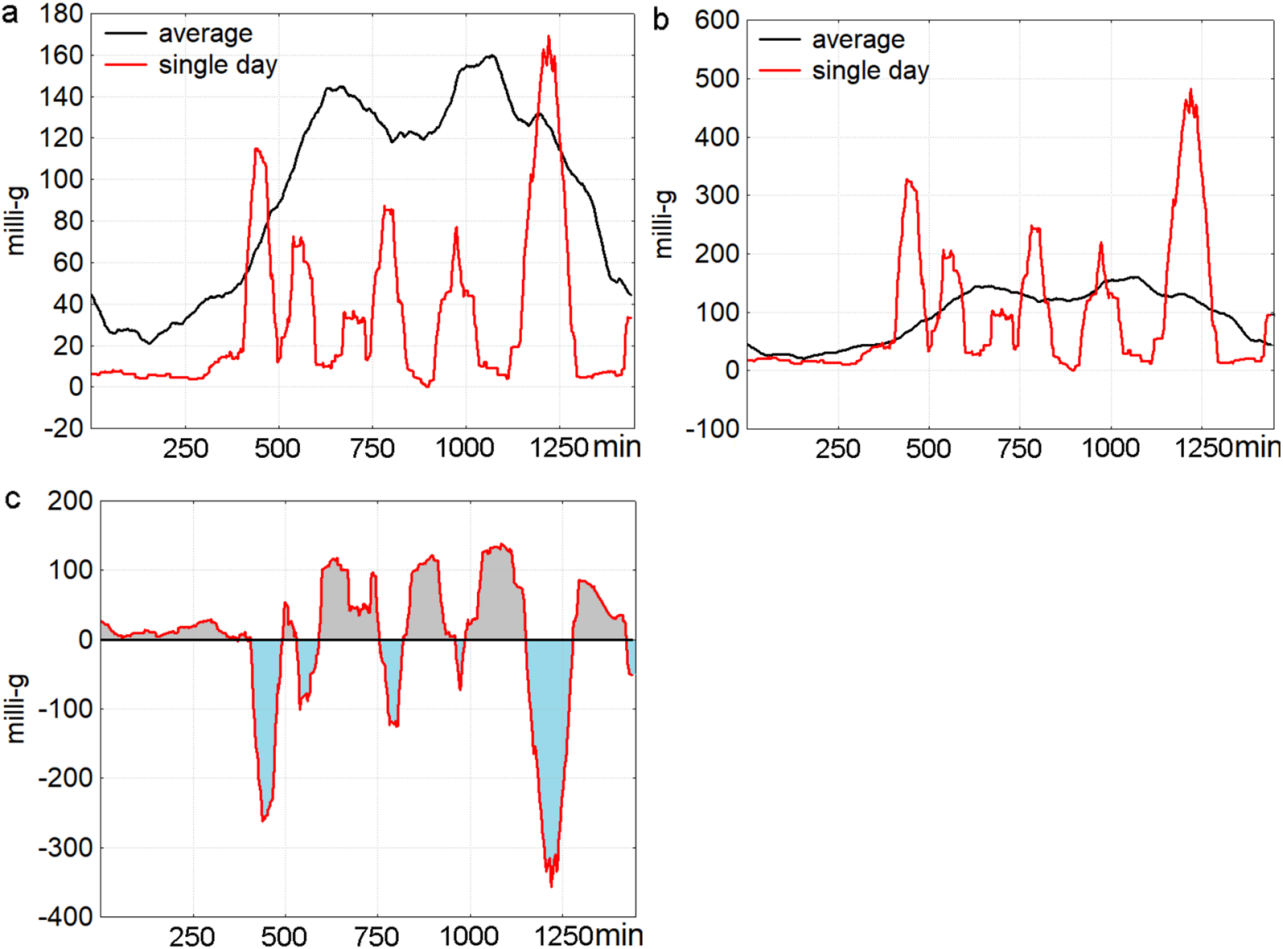
Process of calculating circadian form differences (FormDiff). (**a**) Euthymic vs. Single-Day Movement Pattern. The average movement pattern during euthymic days for a single subject (black line, mean = 93.4 milli-g) is overlaid with the movement pattern for a single day (red line, mean = 32.8 milli-g), highlighting both the lower activity level on that day and distinct deviations in the temporal activity pattern. (**b**) Aligned Patterns: Adjusted Single-Day Activity. To isolate differences in circadian form, the mean activity level of the single day has been adjusted to match the mean activity of euthymic days (93.4 milli-g), aligning both patterns and allowing for a clearer comparison of differences in circadian form independent of activity intensity. (**c**) Differences Between Adjusted and Euthymic Patterns. The difference between the adjusted single-day pattern and the euthymic baseline pattern is shown, with positive deviations shaded in grey and negative deviations in light blue. Since the average of these differences is zero, all negative values are converted to positive, resulting in a FormDiff score of 71.1 milli-g. For better visualization, the graphs have been smoothed using a 60-point moving average

To capture additional circadian characteristics, we included parameters to address the limited sensitivity of IS and IV to the temporal distribution of activity. Circadian activity can be conceptualized as total daily activity – operationalized through parameters like mean daily activity and its variance – and circadian form, which considers temporal patterns. If, for example, patients wake up later and stay up longer, this temporal shift may go undetected if only total activity is analyzed, as the overall daily activity and within-day variance remain unchanged. However, circadian form parameters indicate whether an activity occurs at specific times (e.g., 7:00 a.m. or 3:00 p.m.), which is of major importance because clinical observation suggests that differences in activity in the context of affective episodes are more pronounced at certain times of the day than at others (e.g., morning low during depressive episodes in the context of melancholic features (Parker et al. 2000). Based on Hill (2002), we separated total activity scores from circadian form by calculating daily deviations from each individual’s usual activity pattern, yielding differences in mean parameters (MeanDiff) and differences in form parameters (FormDiff), calculated as follows:

1. Creating a standard circadian pattern: A “minute-by-minute” circadian pattern for each participant was generated by averaging activity for each of the 1440 min of the day across all euthymic days, resulting in 1440 data points (minutes) that represent the average euthymic pattern. This average reflects consistent activity patterns, omitting infrequent activities (those occurring on just one or a few days).
2. Mean activity differences (MeanDiff): Minute-by-minute differences were calculated by subtracting the standard minute-by-minute circadian pattern from each minute of each day. To assess MeanDiff for each day, we averaged these minute-by-minute differences across the 1440 min per day, yielding the average deviation in daily activity from the usual pattern, focusing on total activity without preserving form*.*
3. Circadian form differences (FormDiff): To specifically capture the form of circadian rhythm, we adjusted each minute’s activity level by normalizing it to the average activity observed during euthymic days: Minute_day_adj_ = Minute_day_ x Mean_average_/Mean_day_. This adjustment is necessary because, for instance, a patient experiencing a manic episode may exhibit increased activity intensity per minute while still maintaining the usual circadian pattern (e.g., similar wake and sleep times). By adjusting the activity level to match the average euthymic activity, we could isolate changes in circadian form without the confounding effects of overall activity intensity. This process is shown in Figs. 1a and 1b, which show activity patterns before and after the adjustment for total activity. Once adjusted, we calculated the minute-by-minute differences between the euthymic baseline pattern and each individual day’s activity (Fig. 1c). To ensure these deviations reflect only changes in form (e.g., shifts in peak activity times), any negative values in these minute-by-minute differences were converted to positive values. These adjusted values were then averaged across all 1440 min of the day, producing a daily FormDiff score. FormDiff, therefore, quantifies deviations in the timing or structure of activity peaks, capturing any shifts or variations in the pattern of daily activity. Finally, FormDiff and MeanDiff scores were averaged separately across all depressive days, (hypo)manic days, and euthymic days.
4. Days with > 360 min of nonwear times or low mean activity (< 20 milli-g; 15 days) were excluded, resulting in 2669 days retained for analysis. Nonwear intervals were set to zero to reduce sensor noise (Hill 2002).

### Statistical analysis

Our analyses were based on multilevel models (mixed models) with random intercepts to account for the hierarchical structure, with individual data points nested within participants. First, we fitted generalized linear mixed models for each circadian rhythm measure to predict depressive days (yes/no; depressive vs. euthymic episode) and (hypo)manic days (yes/no; (hypo)manic vs. euthymic episode) as binary dependent variables (logit models). Second, we employed separate general linear mixed models to examine the effects of circadian rhythm measures on the two dimensional (latent) outcome variables “depressive” and “(hypo)manic” (linear models). To account for potential confounding effects, the variables age, sex, and minutes of nonwear time per day were included as covariates in each model. Moreover, all momentary predictors were centered on their respective person means in all models. Receiver operating characteristic (ROC) analyses were performed on all logistic mixed-effects models to evaluate classification performance, with area under the curve (AUC) values reported. All statistical analyses were conducted in Julia (version 1.11) using the MixedModels.jl package for linear and logistic mixed-effects models.

## Results

### Sample characteristics

27 outpatients participated in this study over the course of one year, contributing a total of 9,836 days of data (see Table 1 for an overview of sample characteristics). Of these, physical activity data obtained from acceleration sensors were available for 4,055 days, with individual contributions ranging from 4 to 336 days per participant. Reasons for data gaps included sensor transition-related data loss, periods of sensor discharge, delays in data transfer due to memory capacity limits, device malfunctions (e.g., non-waterproof sensors), and reduced compliance with wearing sensors during summer months. Four participants lacked sufficient activity data and were excluded from all analyses. To ensure adequate circadian pattern information, we further excluded days with more than 360 min of nonwear time, resulting in a final dataset of 2,669 days. In this final data set, individual contributions ranged from 22 to 301 days per participant. The final sample comprised 23 patients (16 female, 7 male; mean age = 46 years, SD = 12.3, age range: 25–70). Among them, 11 participants experienced at least one depressive episode. Specifically, 6 participants experienced one depressive episode, 4 participants experienced two depressive episodes, and one participant experienced three depressive episodes. Additionally, 10 participants experienced at least one (hypo)manic episode. Of these, two participants experienced one hypomanic episode, 7 participants experienced two (hypo)manic episodes, and one participant experienced three episodes. Four of these 17 participants experienced both depressive and (hypo)manic episodes. For the purpose of this analysis, we counted both recurrences and relapses as episodes.

**Table 1:** Sample characteristics

**Characteristic**	N	**M (SD)**	**Range**	**Notes**
Participants	27	-	-	Initial sample size
Days of data contributed	9,836	-	-	Total days of data collected
Days with activity data	4,055	-	4–336	Excluded: Data gaps due to technical or compliance issues
Days with sufficient data	2,669	116.04 (77.23)	22–301	After excluding days with > 360 min nonwear time
Final participants	23	-	-	4 excluded for insufficient activity data
Sex (female/male)	16/7	-	-	Final sample
Age (years)	-	46 (12.3)	25–70	Final sample
Participants with depressive episodes	9	17.3 (MD)	-	At least one depressive episode
Participants with (hypo)manic episodes	7	14.6 (MD)	-	Two participants experienced both episode types

*MD* = Mean Duration

### Descriptive statistics

**Fig. 2:**
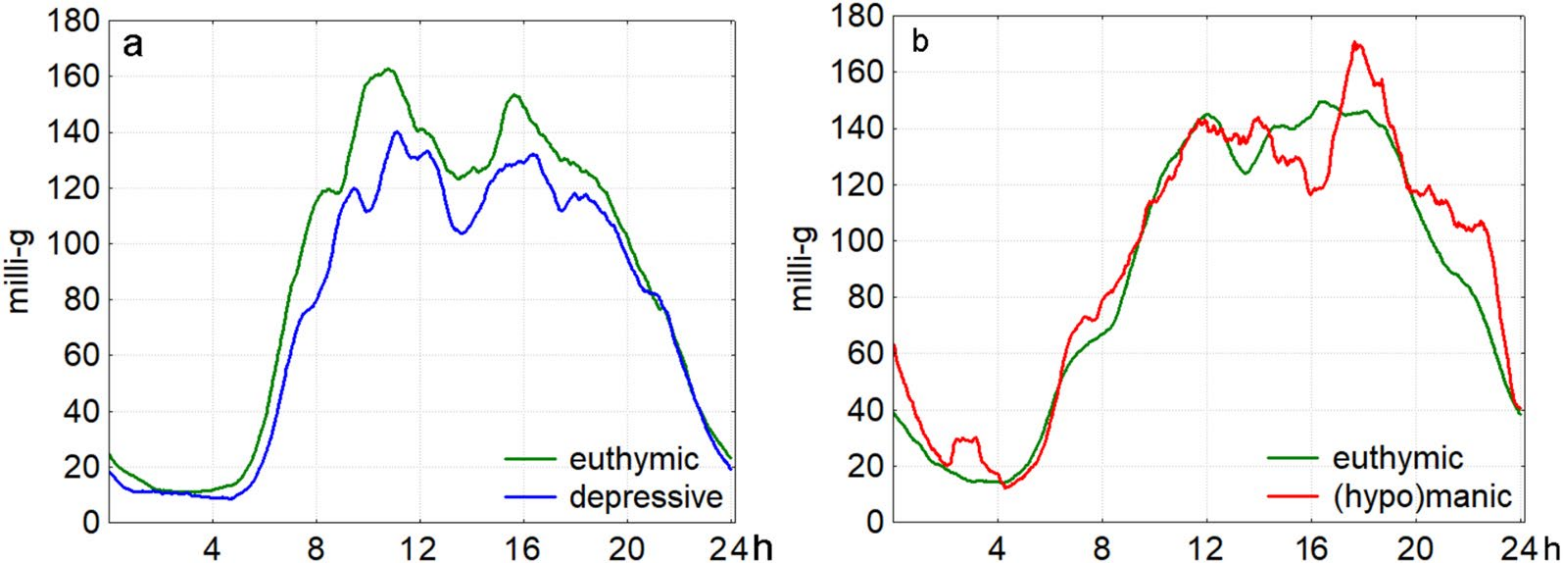
Grand Averages of Activity Patterns Comparing Mood States Across Participants Grand averages of activity patterns across all participants, comparing depressive vs. euthymic days (n = 9, panel a, left) and (hypo)manic vs. euthymic days (n = 7, panel b, right). For better visualization, the graphs have been smoothed using a 60-point moving average

Figure 2 presents the circadian rhythm data, illustrating group differences between euthymic and depressive days (Fig. 2a) and between euthymic and (hypo)manic days (Fig. 2b). Descriptively, mean activity levels were lower on depressive days compared to euthymic days (Fig. 2a) and higher on (hypo)manic days compared to euthymic days (Fig. 2b). Additionally, depressive days exhibited a slight delay in activity onset, while (hypo)manic days showed a tendency toward an earlier onset and extended activity into the evening.

### Multilevel logit prediction of illness episodes vs. euthymia

Table 2 and Table 3 summarize the results from multilevel logit models predicting depressive days (vs. euthymic days) and (hypo)manic days (vs. euthymic days), separate for each circadian rhythm measure. Findings revealed that MeanDiff, FormDiff, and IS significantly predicted depressive episodes, with higher FormDiff values and lower MeanDiff and IS values associated with an increased likelihood of a depressive episode. The covariates age, sex, and nonwear time did not show significant effects in these models.

**Table 2:** Results of multilevel logit models for the categorical (binary) depressive outcome

Depressive						
Models	Variables	*B*	*SE*	*P*	*95% CI*	*OR*
MeanDiff	(Intercept)	−6.86	12.51	0.58	[−12.51, 12.51]	0.00
	MeanDiff	−0.02	0.00	< 0.001	[−0.02, −0.01]	0.98
	Min nonw	−0.00	0.09	1.00	[−0.17, 0.17]	1.00
	Age	−0.02	0.13	0.87	[−0.26, 0.22]	0.98
	Sex	0.01	3.46	0.99	[−6.67, 6.69]	1.01
FormDiff	(Intercept)	−4.63	7.87	0.56	[−7.87, 7.87]	0.00
	FormDiff	0.03	0.01	< 0.001	[0.02, 0.04]	1.03
	Min nonw	−0.00	0.06	0.99	[−0.12, 0.12]	1.00
	Age	−0.02	0.08	0.76	[−0.18, 0.14]	0.98
	Sex	0.08	2.12	0.97	[−3.98, 4.14]	1.08
IS	(Intercept)	−10.61	14.15	0.45	[−14.15, 14.15]	0.00
	IS	−0.80	0.31	0.009	[−1.41, −0.19]	0.45
	Min nonw	0.01	0.10	0.95	[−0.18, 0.20]	1.01
	Age	0.03	0.15	0.83	[−0.26, 0.32]	1.03
	Sex	0.31	3.86	0.93	[−7.28, 7.90]	1.37
IV	(Intercept)	−10.56	13.90	0.45	[−13.90, 13.90]	0.00
	IV	−0.90	1.01	0.38	[−2.88, 1.08]	0.41
	Min nonw	0.01	0.10	0.94	[−0.18, 0.20]	1.01
	Age	0.03	0.15	0.83	[−0.25, 0.31]	1.01
	Sex	0.31	3.79	0.93	[−7.11, 7.73]	1.01
Min. non-wear	(Intercept)	−9.69	6.86	0.16	[−6.86, 6.86]	0.00
	Min. non-wear	−0.00	0.00	0.38	[−0.00, 0.00]	1.00
	Age	0.03	0.14	0.82	[−0.24, 0.30]	1.03
	Sex	0.35	3.53	0.92	[−7.19, 7.89]	1.01

*MeanDiff* = mean difference in activity; *FormDiff* = circadian form difference; *IS* = interdaily stability; *IV* = intradaily variability; *Min. non-wear* = minutes of non-wear time per day; *Age*, *sex* and *min nonw.* are Level 2 variables. *N* = 23 patients; *Number of observations* = 2,375

**Table 3:** Results of multilevel logit models for the categorical (binary) (hypo)manic outcome

(Hypo)manic						
Models	Variables	*B*	*SE*	*P*	*95% CI*	*OR*
MeanDiff	(Intercept)	−8.25	16.21	0.61	[−16.21, 16.21]	0.00
	MeanDiff	0.02	0.01	0.007	[0.01, 0.03]	1.02
	Min nonw	−0.01	0.14	0.95	[−0.27, 0.25]	0.99
	Age	0.02	0.13	0.89	[−0.24, 0.28]	1.02
	Sex	0.12	3.52	0.97	[−6.75, 6.99]	1.13
FormDiff	(Intercept)	−8.36	16.78	0.62	[−16.78, 16.78]	0.00
	FormDiff	−0.02	0.01	0.054	[−0.04, 0.00]	0.98
	Min nonw	−0.01	0.15	0.96	[−0.27, 0.25]	0.99
	Age	0.01	0.14	0.92	[−0.26, 0.28]	1.01
	Sex	0.15	3.65	0.97	[−6.97, 7.27]	1.16
IS	(Intercept)	−10.82	15.63	0.49	[−15.63, 15.63]	0.00
	IS	0.05	0.33	0.88	[−0.57, 0.67]	1.05
	Min nonw	0.01	0.14	0.92	[−0.26, 0.28]	1.01
	Age	0.02	0.13	0.87	[−0.23, 0.27]	1.02
	Sex	0.34	3.45	0.92	[−6.50, 7.18]	1.40
IV	(Intercept)	−9.11	15.73	0.56	[−15.73, 15.73]	0.00
	IV	0.92	0.78	0.24	[−0.62, 2.45]	2.50
	Min nonw	0.00	0.14	0.99	[−0.27, 0.27]	1.00
	Age	0.02	0.13	0.89	[−0.23, 0.27]	1.02
	Sex	0.11	3.43	0.97	[−6.67, 6.89]	1.00
Min. non-wear	(Intercept)	−9.15	6.28	0.15	[−6.28, 6.28]	0.00
	Min. non-wear	−0.00	0.00	0.06	[−0.00, 0.00]	1.00
	Age	0.02	0.13	0.90	[−0.23, 0.27]	1.02
	Sex	0.16	3.47	0.96	[−6.82, 7.14]	1.18

*MeanDiff* = mean difference in activity; *FormDiff* = circadian form difference; *IS* = interdaily stability; *IV* = intradaily variability; *Min. non-wear* = minutes of non-wear time per day; *Age*, *sex* and *min nonw.* are Level 2 variables. *N* = 23 patients; *Number of observations* = 2,321

For (hypo)manic episodes, MeanDiff emerged as a significant predictor, with higher MeanDiff values correlating with an increased likelihood of (hypo)mania. Again, none of the covariates age, sex, and nonwear time reached significance. In two additional models, nonwear time was tested as the primary predictor for depressive and (hypo)manic episodes, respectively, but it did not significantly predict either outcome.

ROC analyses of the logistic models showed excellent classification performance, with AUC values ranging from 0.89 to 0.91 at the day level (see Supplementary Table S1 and Supplementary Figures S1-S10 for detailed results). However, the ROC analyses were based on the same data that were used to fit the models. Future studies should replicate these findings using training/test splits or independent validation datasets.

### Multilevel prediction of dimensional values of depression and mania

We further analyzed the dimensional outcomes for depression and mania using latent factors, as described by Ebner-Priemer et al. (2020), integrating classificatory and dimensional expert ratings along with daily self-reports. In separate models, all circadian rhythm predictors (MeanDiff, FormDiff, IS, and IV) significantly predicted both depression and mania (Table 4 and Table 5). Importantly, effect directions differed: a) They were opposite for depression vs. mania outcomes and b) they were consistent with the directions observed in the logit models. Specifically, higher FormDiff values and lower MeanDiff, IS, and IV values were associated with higher levels of depression. Conversely, lower FormDiff values and higher MeanDiff, IS, and IV values were linked to increased levels of (hypo)mania. The covariates age, sex, and nonwear time did not have significant effects in these models. Nonwear minutes also did not significantly predict either dimension when tested as a primary predictor in separate models.

**Table 4:** Results of multilevel linear mixed models for the dimensional (latent) depressive outcome

Depressive						
Models	Variables	*B*	*SE*	*P*	*95% CI*	*Std. ß*
MeanDiff	(Intercept)	0.26	0.35	0.45	[−42, 0.94]	0.00
	MeanDiff	−0.00	0.00	< 0.001	[−0.00, −0.00]	−0.11
	Min nonw	0.00	0.00	0.72	[−0.00, 0.01]	0.02
	Age	−0.01	0.00	0.07	[−0.01, 0.00]	−0.16
	Sex	0.07	0.09	0.41	[−0.10, 0.24]	0.07
FormDiff	(Intercept)	0.26	0.35	0.45	[−7.87, 7.87]	0.00
	FormDiff	0.00	0.00	< 0.001	[0.02, 0.04]	0.10
	Min nonw	0.00	0.00	0.72	[−0.12, 0.12]	0.02
	Age	−0.01	0.00	0.07	[−0.18, 0.14]	−0.16
	Sex	0.07	0.09	0.41	[−3.98, 4.14]	0.07
IS	(Intercept)	0.26	0.35	0.45	[−0.42, 0.94]	0.00
	IS	−0.09	0.03	0.001	[−0.15, −0.04]	−0.06
	Min nonw	0.00	0.00	0.72	[−0.00, 0.01]	0.02
	Age	−0.01	0.00	0.07	[−0.01, 0.00]	−0.16
	Sex	0.07	0.09	0.41	[−0.10, 0.24]	0.07
IV	(Intercept)	0.26	0.35	0.45	[−0.42, 0.94]	0.00
	IV	−0.28	0.09	0.002	[−0.45, −0.10]	−0.06
	Min nonw	0.00	0.00	0.72	[−0.00, 0.01]	0.02
	Age	−0.01	0.00	0.07	[−0.01, 0.00]	−0.16
	Sex	0.07	0.09	0.41	[−0.10, 0.24]	0.07
Min. non-wear	(Intercept)	0.37	0.16	0.02	[0.06, 0.67]	0.00
	Min. non-wear	−0.00	0.00	0.39	[−0.00, 0.00]	−0.02
	Age	−0.01	0.00	0.06	[−0.01, 0.00]	−0.16
	Sex	0.08	0.09	0.39	[−0.10, 0.25]	0.07

*MeanDiff* = mean difference in activity; *FormDiff* = circadian form difference; *IS* = interdaily stability; *IV* = intradaily variability; *Min. non-wear* = minutes of nonwear time per day; *Age*, *sex* and *min nonw.* are Level 2 variables. *N* = 23 patients; *Number of observations* = 2,537

**Table 5:** Results of multilevel linear mixed models for the dimensional (latent) (hypo)manic outcome

(Hypo)manic						
Models	Variables	*B*	*SE*	*P*	*95% CI*	*Std. ß*
MeanDiff	(Intercept)	−0.04	0.09	0.64	[−0.21, 0.13]	−0.00
	MeanDiff	0.00	0.00	< 0.001	[0.00, 0.00]	0.10
	Min nonw	−0.00	0.00	0.36	[−0.00, 0.00]	−0.03
	Age	0.00	0.00	0.01	[0.00, 0.00]	0.09
	Sex	−0.03	0.02	0.08	[−0.07, 0.00]	−0.06
FormDiff	(Intercept)	−0.04	0.09	0.63	[−0.21, 0.31]	−0.00
	FormDiff	−0.00	0.00	0.001	[−0.00, −0.00]	−0.07
	Min nonw	−0.00	0.00	0.36	[−0.00, 0.00]	−0.03
	Age	0.00	0.00	0.01	[0.00, 0.00]	0.09
	Sex	−0.03	0.02	0.08	[−0.07, 0.00]	−0.06
IS	(Intercept)	−0.04	0.09	0.63	[−21, 0.13]	−0.00
	IS	0.04	0.02	0.024	[0.01, 0.07]	0.04
	Min nonw	−0.00	0.00	0.36	[−0.00, 0.00]	−0.03
	Age	0.00	0.00	0.01	[0.00, 0.00]	0.09
	Sex	−0.03	0.02	0.08	[−0.07, 0.00]	−0.06
IV	(Intercept)	−0.04	0.09	0.64	[−0.21, 0.13]	−0.00
	IV	0.20	05	< 0.001	[0.09, 0.30]	0.07
	Min nonw	−0.00	0.00	0.36	[−0.00, 0.00]	−0.03
	Age	0.00	0.00	0.01	[0.00, 0.00]	0.09
	Sex	−0.03	0.02	0.08	[−0.07, 0.00]	−0.06
Min. non-wear	(Intercept)	−0.11	0.04	0.002	[−0.19, −0.04]	−0.00
	Min. non-wear	−0.00	0.00	0.30	[−0.00, 0.00]	−0.02
	Age	0.00	0.00	0.01	[0.00, 0.00]	0.09
	Sex	−0.03	0.02	0.10	[−0.07, 0.01]	−0.06

*MeanDiff* = mean difference in activity; *FormDiff* = circadian form difference; *IS* = interdaily stability; *IV* = intradaily variability; *Min. non-wear* = minutes of nonwear time per day; *Age*, *sex* and *min nonw.* are Level 2 variables. *N* = 23 patients; *Number of observations* = 2,532

### Exploratory longitudinal analyses

**Fig. 3:**
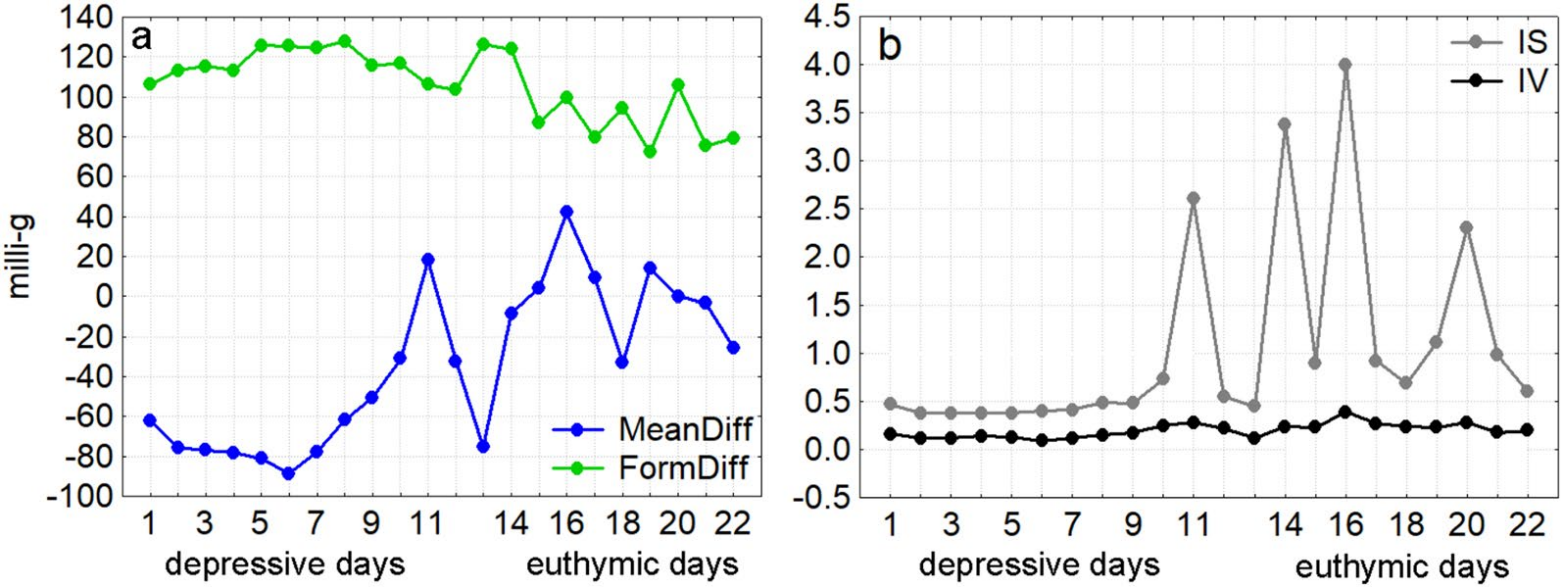
Circadian rhythm parameter changes over 22 days for a single participant

Recognizing the potential of digital phenotyping in BD for real-time episode prediction, we conducted an exploratory analysis of a single participant’s circadian rhythm parameters over three weeks, capturing a transition from depression to euthymia. Figure 3 depicts the daily trajectories of MeanDiff, FormDiff, IS, and IV during this period, illustrating the potential utility of these parameters for identifying prodromal periods in future research.

Days 1–11 correspond to a depressive episode, days 14–22 to an euthymic state, while days 12–13 lack clinical ratings.(**a**) (left). Mean and Form Differences in Activity Patterns on Depressive vs. Euthymic Days. The blue line depicts the mean activity difference (MeanDiff), which is notably lower on depressive days (−60.4 vs. 0 milli-g), indicating reduced mean activity compared to euthymic days. In contrast, the green line represents the form difference (FormDiff), which is elevated during depressive days (117.4 vs. 90.9 milli-g), indicating greater deviations from the participant’s usual daily rhythm. The two parameters show a negative correlation (r = −0.67) (**b**) (right). Interdaily Stability and Variability Across Mood States. The grey line represents interdaily stability (IS), which is higher and more variable during euthymic days (1.65 vs. 0.65), indicating a more consistent daily rhythm in the euthymic state. The black line displays intradaily variability (IV), which is slightly reduced on depressive days (0.16 vs. 0.25), suggesting fewer shifts between active and inactive states within each day.

## Discussion

This study examined the potential of circadian movement parameters to differentiate between euthymic, depressive, and (hypo)manic episodes in individuals with BD. Although the study was explorative by nature, across various analytical approaches, our findings consistently revealed distinct circadian patterns associated with depressive and (hypo)manic states, underscoring the clinical relevance of circadian rhythm disruptions in BD.

Our analyses indicated that the likelihood of a depressive episode or day increased with lower overall daily activity (MeanDiff), reduced daily rhythm fragmentation (IV), decreased interdaily stability (IS), and a more consistent circadian rhythm structure (FormDiff). When depression was modeled as a latent variable – integrating both biweekly expert ratings and daily self-ratings – all circadian predictors reached significance, while in the categorical outcome models, only IS, MeanDiff, and FormDiff were statistically significant predictors. Conversely, the models predicting (hypo)manic episodes showed an inverse pattern: Higher daily activity (MeanDiff), increased daily rhythm fragmentation (IV), higher interdaily stability (IS), and a less structured circadian rhythm (FormDiff) were all associated with (hypo)mania. In the latent models for (hypo)mania, all predictors were statistically significant, while MeanDiff alone reached significance in the categorical models. These findings suggest that circadian movement parameters can reliably differentiate mood states in BD, with circadian rhythm disruptions serving as important clinical markers.

Further examination of individual parameters strengthens these observations. As expected, higher total activity levels (MeanDiff) were associated with (hypo)manic states, while lower activity levels correlated with depressive states, aligning with well-established clinical profiles. Lower activity levels during depressive episodes reflect core symptoms such as lack of motivation, social withdrawal, and reduced participation in daily activities, potentially serving as an objective marker for depressive states (De Leeuw et al. 2023; Minaeva et al. 2020; Spulber et al. 2022). In contrast, the higher activity levels observed in (hypo)manic episodes indicate increased drive, hyperactivity, and reduced sleep duration, clinically manifesting as excessive energy, impulsive behavior, and more intense engagement in social and working life (Mir et al. 2022; Perry et al. 2016). Similarly, low daily activity (MeanDiff), reduced variability (IV), as well as more stable and rigid circadian patterns might represent objective markers for the reduced energy, psychomotor slowing, and lack of flexibility in daily routines that are characteristic of depressive episodes according to the ICD-11 (Harrison et al. 2021).

Reduced IS, indicative of a weakened circadian rhythm, was linked to depressive episodes, supporting clinical descriptions of depression that include diminished daytime activity and extended rest periods, leading to smaller day-night activity differences. This pattern may be attributed to reduced drive, passivity, and social withdrawal, as well as core symptoms of depression, such as joylessness, loss of interest, and depressed mood (American Psychiatric Association, 2018; Ho et al. 2024; Liao et al. 2025). Conversely, higher IS was linked to a higher level of the continuous (hypo)-manic episodes, reflecting a stronger daily rhythm amplitude, likely driven by heightened daytime activity levels typical of manic stated. This pattern might correspond to the increased daytime activity characteristic of manic states, reflecting heightened drive, pronounced restlessness, and impulsive behavior typically observed during such episodes. Clinically, this would align with the classic representation of mania, characterized by excessively high energy levels, significantly reduced sleep requirements, and an overall intensified daily rhythm, which would allow for a clear distinction from depressive episodes (Dailey & Saadabadi 2024; De Crescenzo et al. 2017).

Rhythm fragmentation (IV) was also related to higher levels of (hypo-)mania. Higher IV values, indicating frequent shifts between active and inactive periods (Gonçalves et al. 2014; Scott et al. 2017; Witting et al. 1990), were associated with higher levels of (hypo-)mania, which often involve increased drive, impulsivity, and prolonged activity periods, including nighttime activity due to reduced sleep needs (Dailey & Saadabadi 2024; Perry et al. 2016). In contrast, lower IV values, indicating a more stable rhythm, correlated with higher depressive symptoms, supporting existing clinical observations, that might reflect the reduced flexibility and consistently low activity levels typical of depression, characterized by diminished drive, withdrawal from daily activities, and a lack of engagement in social and occupational routines (American Psychiatric Association, 2018; McCarthy et al. 2022).

Finally, the FormDiff parameter, representing circadian structure rigidity, was likewise meaningful: Higher- FormDiff (suggesting a more rigid daily rhythm) correlated with depressive days or episodes, whereas lower FormDiff (suggesting a more flexible rhythm) was associated with higher levels of (hypo-)mania, possibly reflecting the impulsive and spontaneous activity patterns typical of mania. Clinically, the increased rigidity of the circadian rhythm (high FormDiff value) observed in depressive episodes might reflect the limited adaptability and reduced flexibility in daily life typical of depressive states (Palagini et al. 2022). This rigidity might represent the psychomotor retardation, diminished drive, and withdrawal tendencies often seen in depression. In contrast, a low FormDiff value, indicating a more flexible rhythm, could reflect the impulsive and spontaneous activity typical of manic episodes (Jakobsen et al. 2022). Manic states are characterized by heightened daytime activity, unpredictable shifts between tasks, and reduced rest periods. This flexibility in circadian patterns might therefore express the hyperactivity, increased drive, and impulsivity that are clinically central to mania (American Psychiatric Association 2018; Harrison et al. 2021; McCarthy et al. 2022; Patapoff et al. 2022; Titone et al. 2022).

A comparison between categorical and latent models in our study revealed that the latent model, which integrates expert ratings with daily data, captured both state transitions and symptom intensity more effectively than the categorical model. Although latent models may not represent the ground truth of BD psychopathology, they offer a more nuanced view of symptomatology and improve the dimensionality and temporal precision of our outcomes.

The study’s key strengths include its 12-month duration, allowing a sufficient number of episodes to occur, and high-frequency assessments integrating expert and self-ratings as well as digital phenotypes with high validity and time-sensitive indices. However, several limitations should be noted. First, while this dataset likely includes one of the highest numbers of labeled days per patient, data availability of the wearable data was tremendously reduced by nonwear time and technical issues. Additionally, for studies focussing on episode prevention, even longer study durations may be advisable, as in our current 18-month RCT (Mühlbauer et al. 2018). Second, the frequent psychopathological assessments employed in this study may have influenced episode prevention, with biweekly interviews and daily ratings potentially acting as an intervention in themselves. Nevertheless, we observed more affective episodes across the 12-month period than initially expected based on patients lifetime histories (estimated incidence of 0.3 depressive, 0.1 hypomanic, and 0.1 manic episodes per year per participant, assuming onset at age 20). Third, missing data and instances of nonwear time were substantial, raising questions about whether lifestyle devices could improve compliance rates. However, such devices typically use varying algorithms and store data externally, which may present legal and regulatory challenges, particularly in Germany. Fourth, given the relatively small sample size, we cannot exclude that some findings may be sample-specific. While our analyses followed classical statistical modelling approaches without cross-validation or bootstrapping, we applied careful model specification and diagnostics to reduce the risk of overfitting. Future studies with larger samples and complementary validation techniques are warranted to further assess the generalizability of these results. Fifth, Activity energy expenditure can be assessed most accurately using doubly labeled water (DLW) (Pontzer et al. 2021), which is currently considered the gold standard for use in free-living conditions. However, due to its high cost, laboratory requirements, and limited temporal resolution, accelerometry is more widely employed in repeated-measures designs. Sixthly, in addition to the clear limitations, there are other possibilities for deriving circadian rhythm indices. For example, one could investigate the most and least active hours (Hennion et al. 2024), perform transfer entropy analysis (Song et al. 2024), or use circadian phase Z scores (Lim et al. 2024). Seventhly, and again an upcoming possibilty, are studies using less burdensome devices, such as rings (Ortiz et al. 2025) to optimize the balance between long-assessment period, data availability and patient burden.

## Conclusions

Our study highlights that circadian movement parameters are valuable tools for distinguishing mood states in BD, underscoring the potential of circadian disruptions as clinical markers for mood episode transitions. While longer monitoring periods and further methodological refinements may enhance predictive accuracy, the integration of high-frequency, multimodal assessments presents a promising approach to deepening our understanding of mood disorder dynamics. Future research with larger samples and extended study durations could clarify the role of circadian rhythms in both mood state identification and episode prevention.

## Supplementary Information

Additional file1

## Data Availability

Data sharing is not applicable at this stage of the study process, as many research questions are not analysed yet. After final publication of additional research questions, the datasets analysed will be available from the corresponding author on reasonable request.

## Abbreviations

BD: Bipolar disorders
DSM: Diagnostic and Statistical Manual of Mental Disorders
HAMD: Hamilton Depression Rating Scale
MADRS: Montgomery‑Asberg Depression Rating Scale
YMRS: Young Mania Rating Scale
GPS: Global Positioning System
BRMRS: Bech Rafaelsen Mania Rating Scale
PA: Physical activity
HC: Healthy controls
IS: Inter-daily stability
IV: Intra-daily variability
MeanDiff: Mean differences in activity
FormDiff: Form Differences
SE: Standard error
RCTs: Randomized controlled trials
