# Supplementary material for: Circadian rhythm parameters differentiate euthymic, manic and depressive mood states in bipolar disorders – an explorative pilot study
Source: Int J Bipolar Disord. 2025 Oct 27;13:30. doi: 10.1186/s40345-025-00396-5 (PMC12559485; doi:10.1186/s40345-025-00396-5)
Supplement: Supplementary file 1 — Additional file1 [file 40345_2025_396_MOESM1_ESM.pdf]

Supplemental materials for:

Circadian rhythm parameters differentiate euthymic, manic and depressive mood states in bipolar disorders – a pilot study

Clemens, J.[1*], Schmitz, S.E.[2], Mühlbauer, E.[1], Reinhard, I.[4], Bauer, M.[1], Neubauer, A.B.[6], Ritter, P.[1,7], Ludwig, V.M. [1], Severus, W.E.[1,3] & Ebner-Priemer, U.W.[2,5]

[1]Department of Psychiatry and Psychotherapy, Faculty of Medicine and University Hospital Carl Gustav Carus, Dresden University of Technology, Dresden, Germany

[2]Mental mHealth Lab, Institute of Sport and Sport Sciences, Karlsruhe Institute of Technology, Karlsruhe, Germany.

[3]Asklepios Klinik Nord - Ochsenzoll, Hamburg, Germany.

[4]Department of Biostatistics, Central Institute of Mental Health, University of Heidelberg, Medical Faculty Mannheim, Mannheim, Germany.

[5]Department of Psychiatry and Psychotherapy, Central Institute of Mental Health, University of Heidelberg, Medical Faculty Mannheim, Mannheim, Germany.

[6]Institute of Psychology, RWTH Aachen University, Aachen, Germany

[7]Institute of Psychiatry, Psychology & Neuroscience (IoPPN), King's College London, UK

Table S1. AUC values for all logistic models based on activity data.

Logistic models	AUC values
**Depressive**	
MeanDiff, min. non-wear, age, gender	0.9091861083186005
FormDiff, min. non-wear, age, gender	0.9009689049121226
IS, min. non-wear, age, gender	0.9097292034989196
IV, min. non-wear, age, gender	0.8952540006538893
Min. non-wear, age, gender	0.9031167307981188
**(Hypo)manic**	
MeanDiff, min. non-wear, age, gender	0.8934977776599599
FormDiff, min. non-wear, age, gender	0.896859581007846
IS, min. non-wear, age, gender	0.9006662601949297
IV, min. non-wear, age, gender	0.8968538033995447
Min. non-wear, age, gender	0.8955831543974057

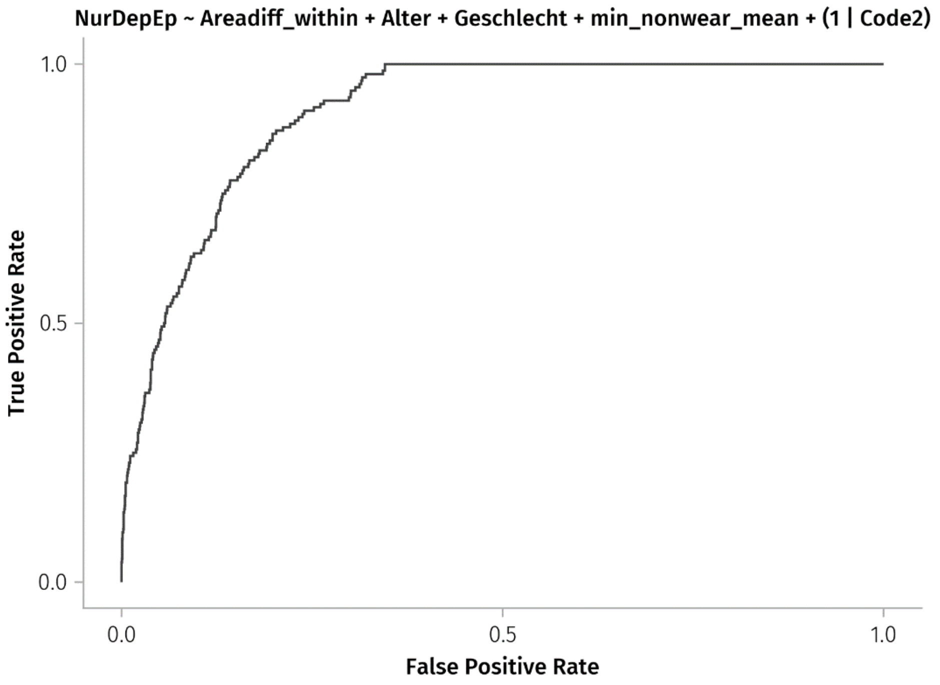

Figure S1. ROC curve for the logistic mixed-effects model predicting depressive episodes based on mean activity differences (Areadiff_within resp. MeanDiff), age, gender, and non-wear time. The curve illustrates the trade-off between sensitivity (true positive rate) and 1 – specificity (false positive rate) across all possible classification thresholds. The area under the curve (AUC) quantifies the model's overall ability to discriminate between depressive and euthymic days.

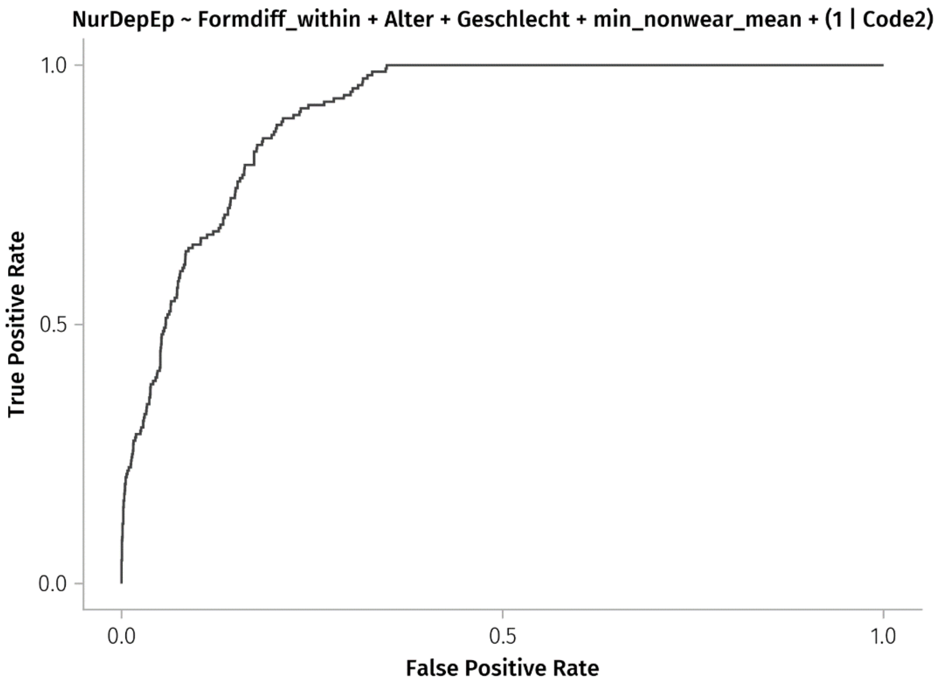

Figure S2. ROC curve for the logistic mixed-effects model predicting depressive episodes based on circadian form differences (Formdiff_within), age, gender, and non-wear time. The curve illustrates the trade-off between sensitivity (true positive rate) and 1 – specificity (false positive rate) across all possible classification thresholds. The area under the curve (AUC) quantifies the model's overall ability to discriminate between depressive and euthymic days at the day level.

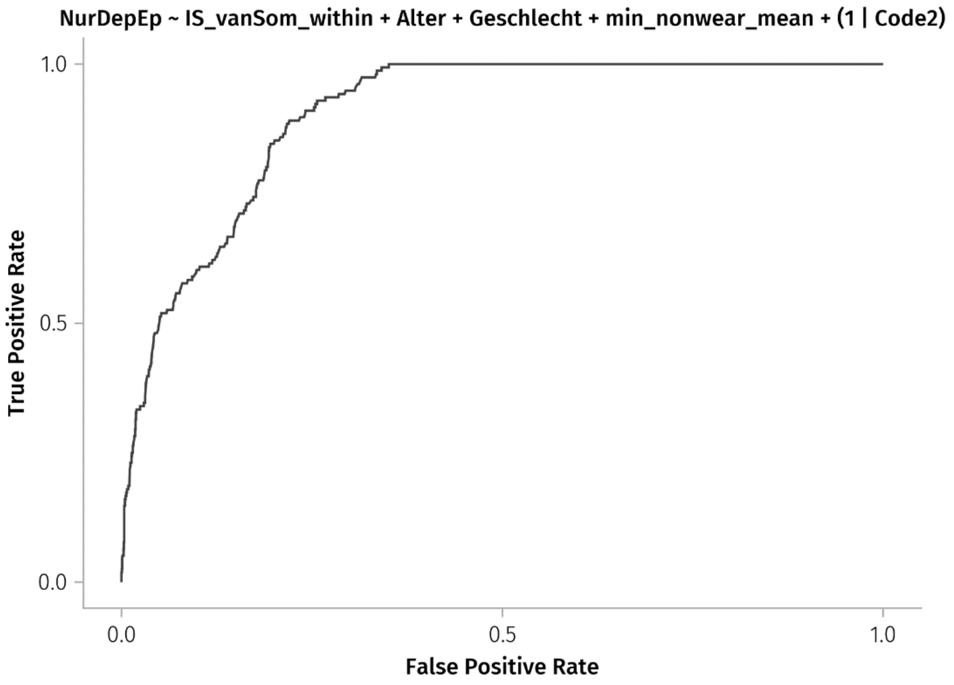

Figure S3. ROC curve for the logistic mixed-effects model predicting depressive episodes based on differences in interdaily stability (IS_vanSom_within), age, gender, and non-wear time. The curve illustrates the trade-off between sensitivity (true positive rate) and 1 – specificity (false positive rate) across all possible classification thresholds. The area under the curve (AUC) quantifies the model's overall ability to discriminate between depressive and euthymic days at the day level.

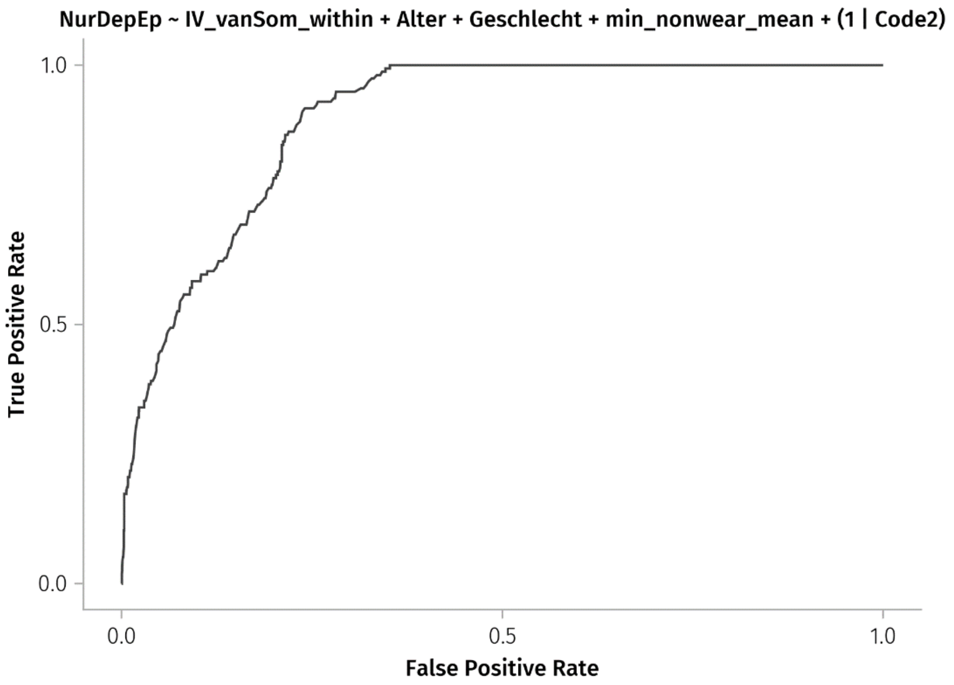

Figure S4. ROC curve for the logistic mixed-effects model predicting depressive episodes based on differences in intradaily variability (IV_vanSom_within), age, gender, and non-wear time. The curve illustrates the trade-off between sensitivity (true positive rate) and 1 – specificity (false positive rate) across all possible classification thresholds. The area under the curve (AUC) quantifies the model's overall ability to discriminate between depressive and euthymic days at the day level.

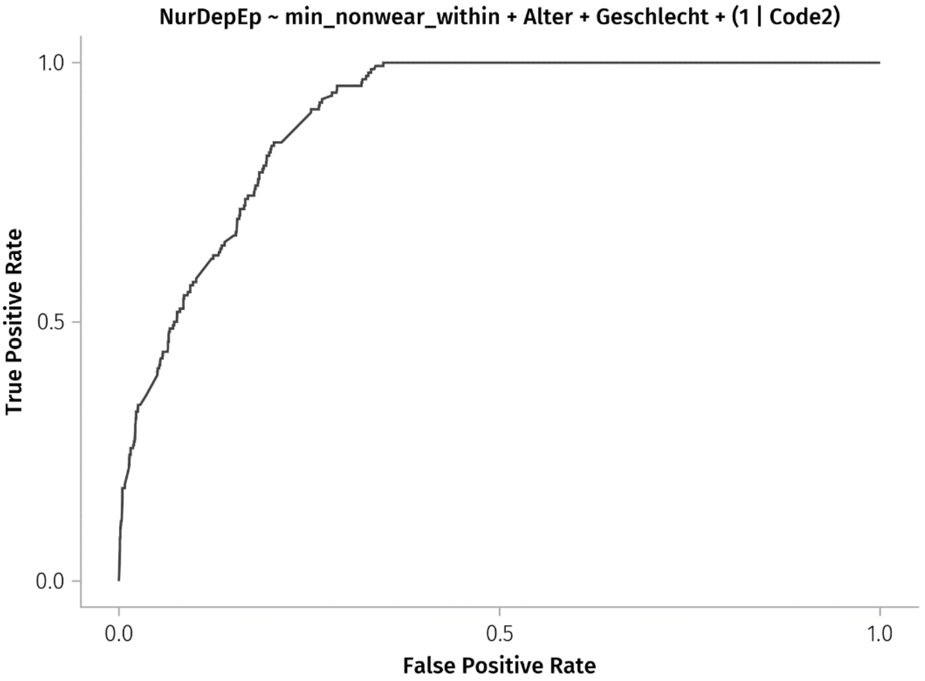

Figure S5. ROC curve for the logistic mixed-effects model predicting depressive episodes based on within-person variation in non-wear time, age, and gender. The curve illustrates the trade-off between sensitivity (true positive rate) and 1 – specificity (false positive rate) across all possible classification thresholds. The area under the curve (AUC) quantifies the model's overall ability to discriminate between depressive and euthymic days at the day level.

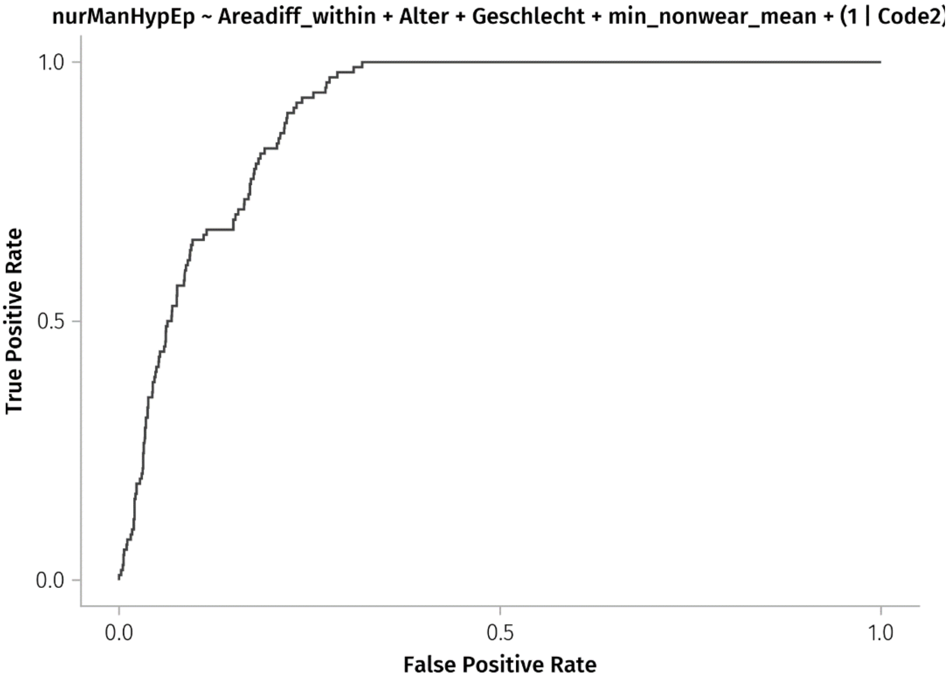

Figure S6. ROC curve for the logistic mixed-effects model predicting (hypo)manic episodes based on mean activity differences (Areadiff_within resp. MeanDiff), age, gender, and non-wear time. The curve illustrates the trade-off between sensitivity (true positive rate) and 1 – specificity (false positive rate) across all possible classification thresholds. The area under the curve (AUC) quantifies the model's overall ability to discriminate between (hypo)manic and euthymic days at the day level.

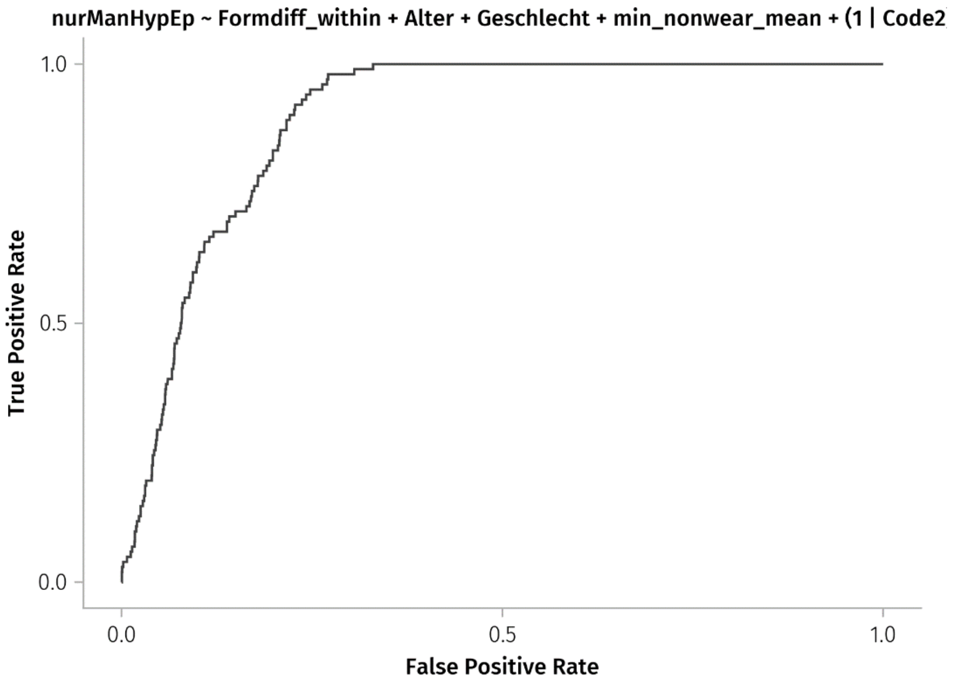

Figure S7. ROC curve for the logistic mixed-effects model predicting (hypo)manic episodes based on circadian form differences (Formdiff_within), age, gender, and non-wear time. The curve illustrates the trade-off between sensitivity (true positive rate) and 1 – specificity (false positive rate) across all possible classification thresholds. The area under the curve (AUC) quantifies the model's overall ability to discriminate between (hypo)manic and euthymic days at the day level.

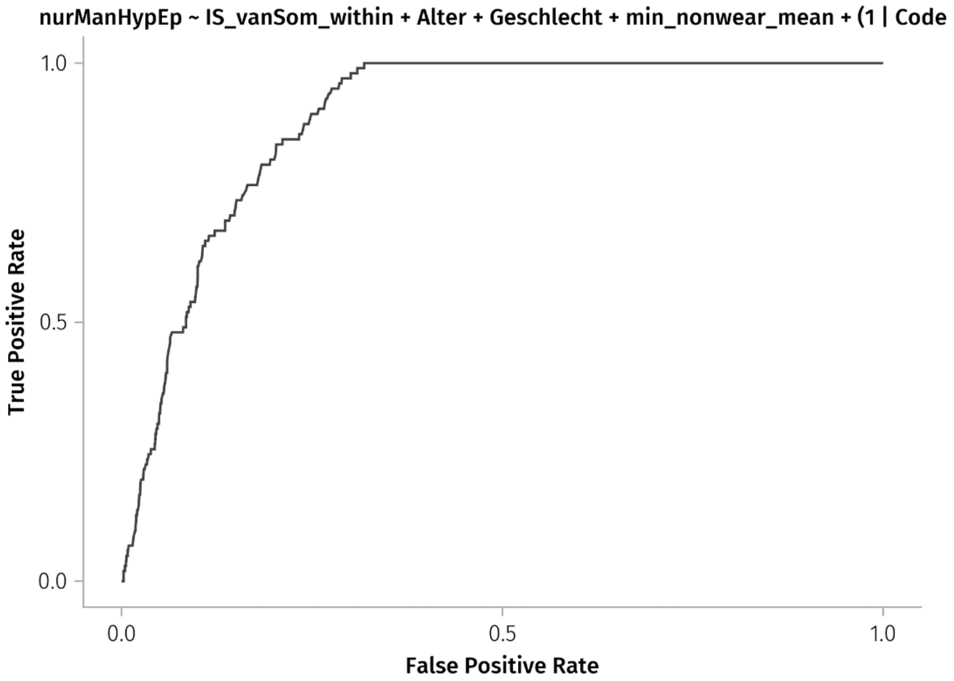

Figure S8. ROC curve for the logistic mixed-effects model predicting (hypo)manic episodes based on differences in interdaily stability (IS_vanSom_within), age, gender, and non-wear time. The curve illustrates the trade-off between sensitivity (true positive rate) and 1 – specificity (false positive rate) across all possible classification thresholds. The area under the curve (AUC) quantifies the model's overall ability to discriminate between (hypo)manic and euthymic days at the day level.

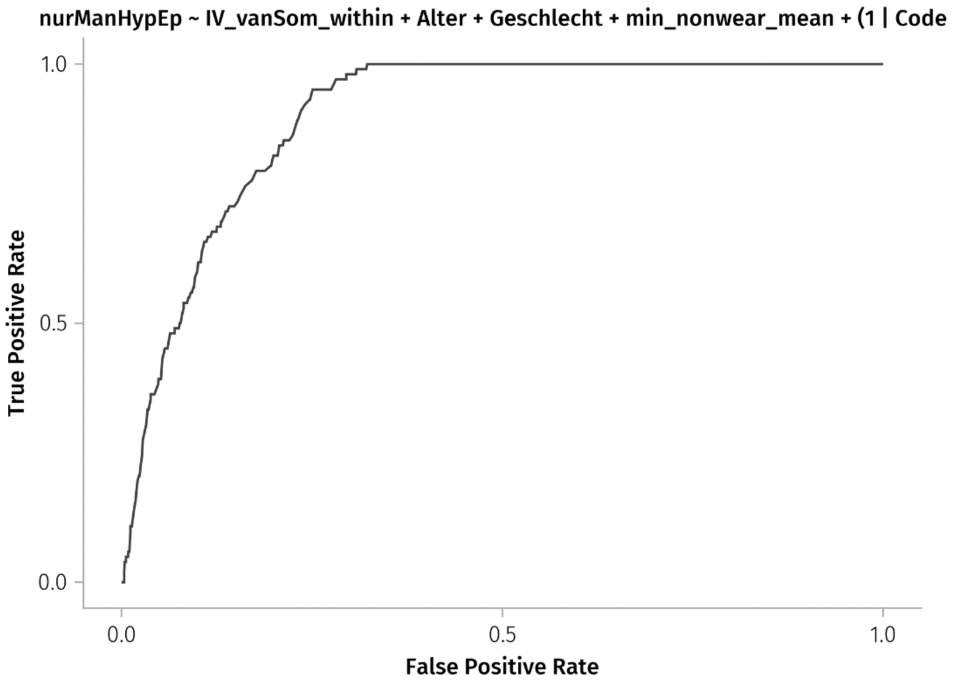

Figure S9. ROC curve for the logistic mixed-effects model predicting (hypo)manic episodes based on differences in intradaily variability (IV_vanSom_within), age, gender, and non-wear time. The curve illustrates the trade-off between sensitivity (true positive rate) and 1 – specificity (false positive rate) across all possible classification thresholds. The area under the curve (AUC) quantifies the model's overall ability to discriminate between (hypo)manic and euthymic days at the day level.

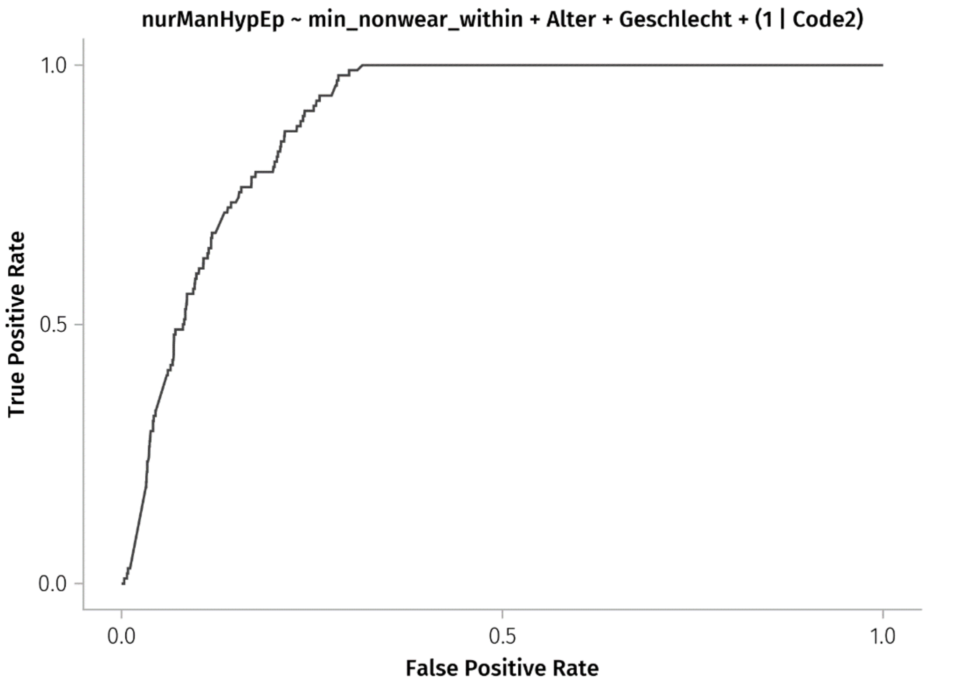

Figure S10. ROC curve for the logistic mixed-effects model predicting (hypo)manic episodes based on within-person variation in non-wear time, age, and gender. The curve illustrates the trade-off between sensitivity (true positive rate) and 1 – specificity (false positive rate) across all possible classification thresholds. The area under the curve (AUC) quantifies the model's overall ability to discriminate between (hypo)manic and euthymic days at the day level.
